# Pneumocephalus With Stroke-Like Symptoms: A Rare Complication of Mastoiditis

**DOI:** 10.7759/cureus.40307

**Published:** 2023-06-12

**Authors:** Shivaan C Oomrigar, Raina K Patel, Robert P Mas, Jose C Fernandez, Daniel I Zapata

**Affiliations:** 1 Internal Medicine, HCA Florida Kendall Hospital, Miami, USA; 2 Allopathic Medicine, Dr. Kiran C. Patel College of Allopathic Medicine, Nova Southeastern University, Fort Lauderdale, USA; 3 Pulmonary and Critical Care Medicine, HCA Florida Kendall Hospital, Miami, USA

**Keywords:** middle ear disease, ventriculitis, critical care, erosive mastoiditis, stroke-like symptoms, pneumocephalus

## Abstract

Pneumocephalus is defined as the presence of gas or air in the intracranial space and typically arises as a result of neurotrauma. Clinically, pneumocephalus most often presents asymptomatically but may cause headache, nausea, vomiting, and confusion. Pneumocephalus arising from mastoiditis is an unforeseen complication with only a handful of cases reported. We report a case of an elderly male who presented with stroke-like symptoms in the setting of erosive mastoiditis with pneumocephalus.

## Introduction

Pneumocephalus involves air in the epidural, subdural, or subarachnoid space within the brain parenchyma or ventricular cavities [1]. Common etiologies of pneumocephalus include trauma, neoplasm, infection, and surgical intervention. A review of 295 patients with pneumocephalus revealed trauma to be the most common cause, accounting for 74% of cases [2]. Pneumocephalus can rarely present following bacterial meningitis, due to putrefactive gas production by both aerobic and anaerobic bacteria such as *Clostridium perfringens* and *Escherichia coli* [3]. However, only a few cases have been reported secondary to middle ear disease [4,5]. Mastoiditis is typically effectively managed with antibiotic therapy; although rare, incomplete treatment may result in worsening and progressive infection leading to fatal neurological complications [6]. This report aims to discuss pneumocephalus as a rare complication of erosive mastoiditis with an atypical stroke-like presentation.

## Case presentation

**Figure 1:**
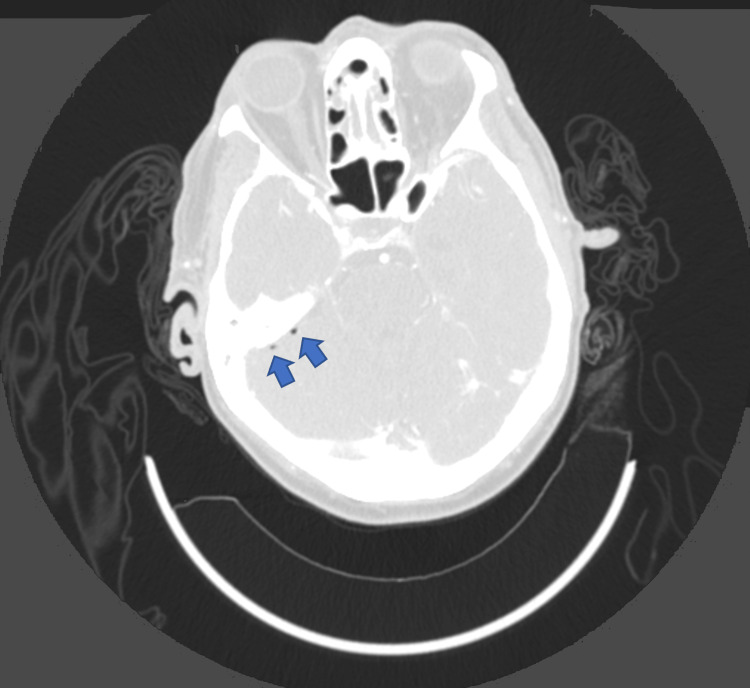
Computed tomography angiography of the head in the axial view demonstrating pneumocephalus along the right tentorium (blue arrows).

**Figure 2:**
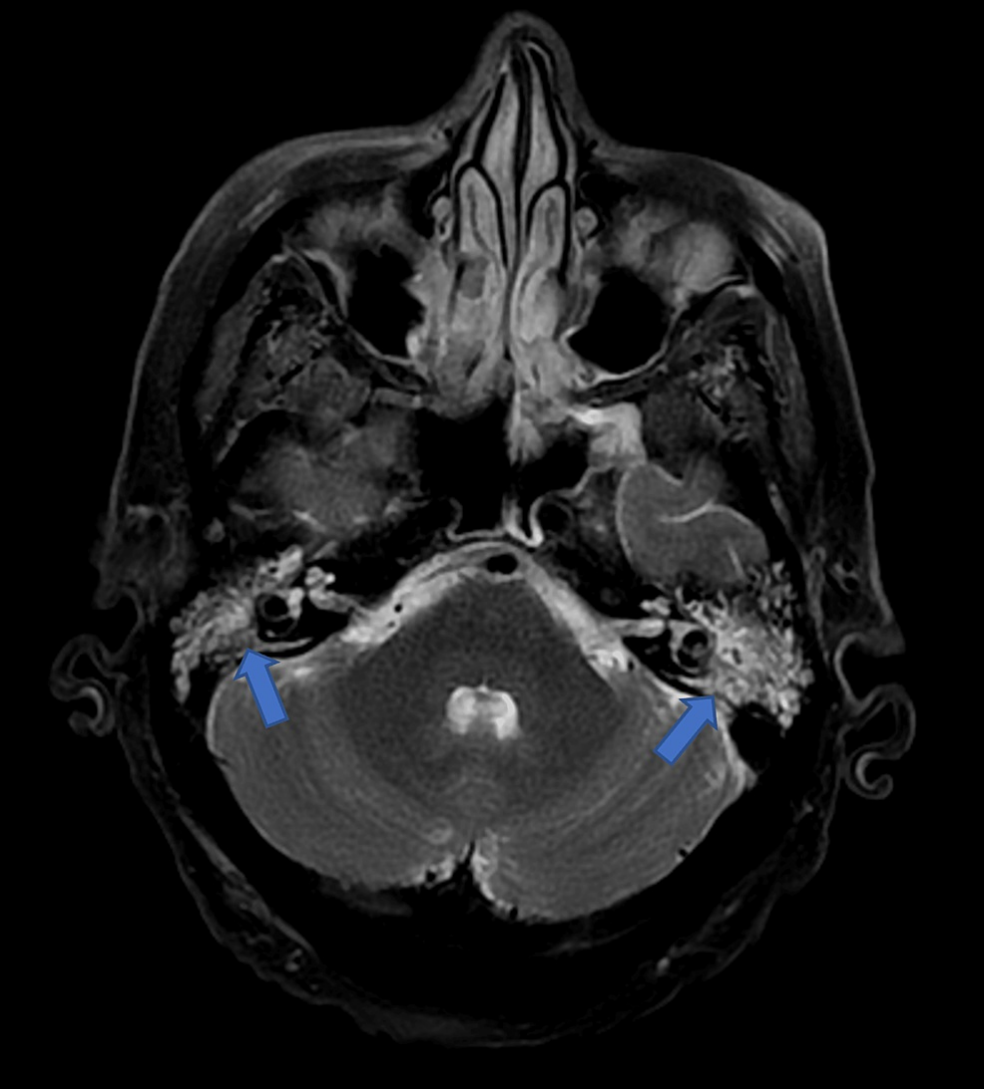
T2-weighted magnetic resonance image of the brain demonstrating bilateral erosive mastoiditis (blue arrows).

An elderly male with no past medical history, no antecedent trauma, and no symptoms prior to the current presentation for admission presented to our facility with new-onset, acute aphasia and dysarthria that began one hour prior to arrival. Initial neurological physical exam was significant for aphasia with no other focal neurological deficits including no motor deficits, no sensory deficits, and no cranial nerve deficits. On arrival, the National Institutes of Health Stroke Scale (NIHSS) score was 6, with deficits of failing to answer the month and age, severely fragmented language aphasia, and severe slurring of words with unintelligible speech. Initial computed tomography (CT) of the brain showed no evidence of an infarct, intracranial hemorrhage, or large vessel occlusion. Therefore, the patient was administered a systemic thrombolytic agent. Further review of the initial CT demonstrated acute bilateral erosive mastoiditis with an intracranial extension with pneumocephalus along the right tentorium cerebelli (Figures 1, 2).

**Figure 3:**
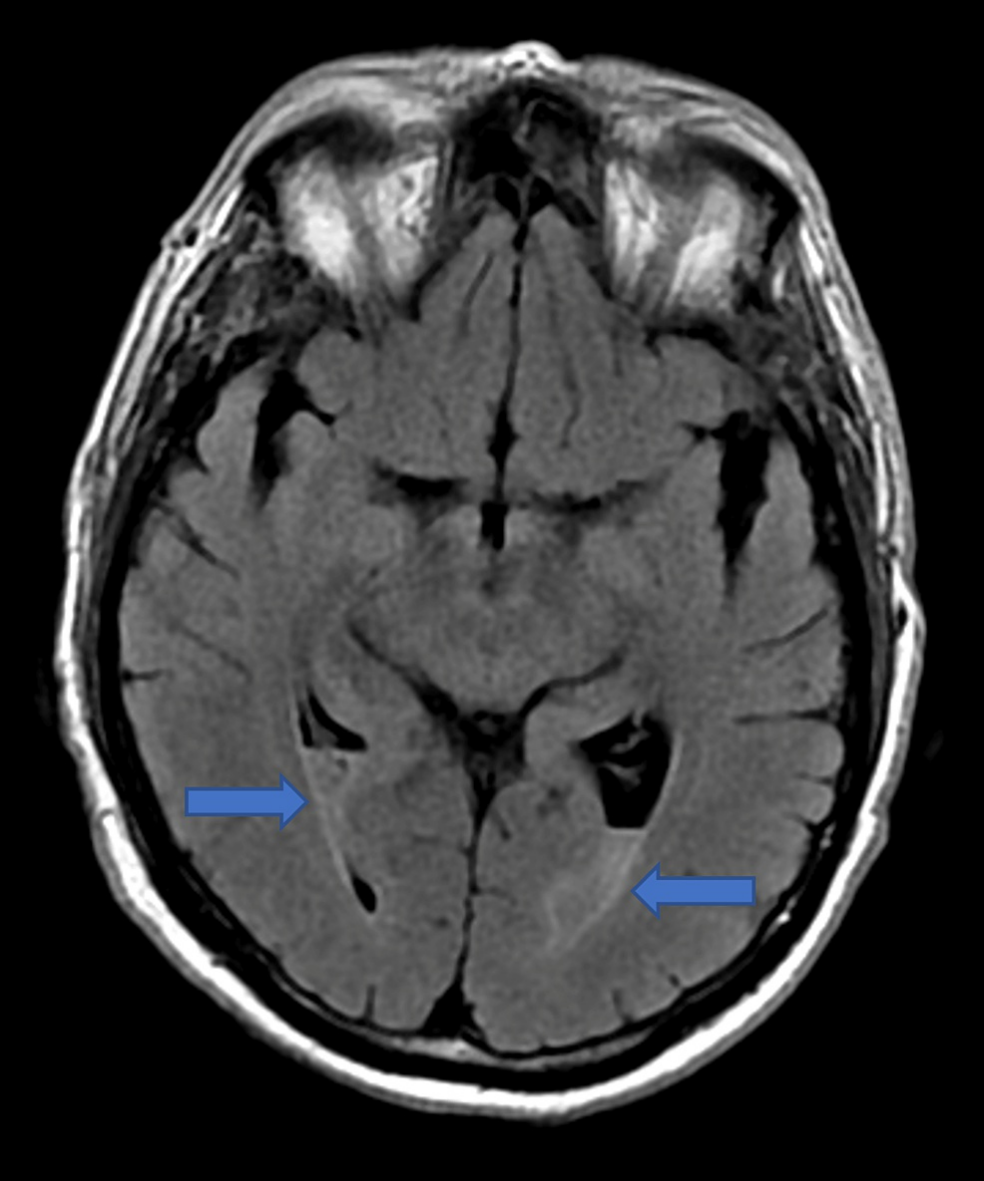
T2 fluid-attenuated inversion recovery (FLAIR) imaging demonstrating layering purulent debris within the bilateral occipital horns (blue arrows).

**Figure 4:**
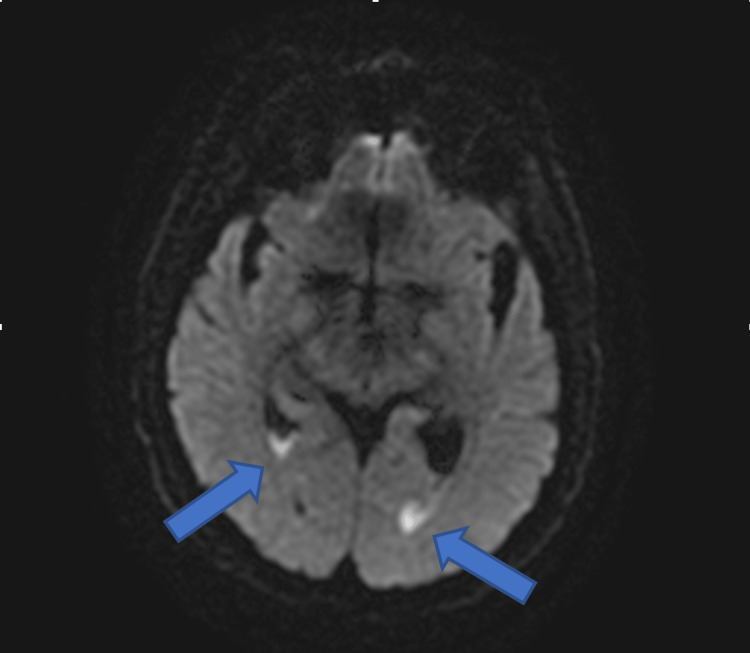
Diffusion-weighted imaging (DWI) demonstrating layering purulent debris within the bilateral occipital horns (blue arrows).

The patient was admitted to the intensive care unit for the management of a presumed acute ischemic stroke and erosive mastoiditis with pneumocephalus. Broad-spectrum intravenous antibiotics were immediately initiated. As a management approach, otolaryngology and neurosurgery recommended a highly specialized procedure including bilateral mastoidectomies. As part of the cerebrovascular event timeline protocol, a magnetic resonance image (MRI) of the brain demonstrated layering purulent debris in the occipital horns of both lateral ventricles representing ventriculitis, given the presence of coalescing mastoiditis and no evidence of a dural sinus thrombosis, acute infarct, intracranial mass, mass effect, or midline shift (Figures 3, 4).

Clinically, the patient slowly improved with the resolution of focal neurological deficits after the initiation of broad-spectrum antibiotics with intravenous vancomycin and piperacillin-tazobactam. Therefore, he did not meet the criteria for an extracranial ventricular drain (EVD) with intrathecal antibiotics or lumbar puncture with cerebrospinal fluid analysis.

However, after an extensive review of the brain imaging and clinical resolution of the neurological symptoms, it was concluded that the neurological presentation was highly due to the mastoiditis and ventriculitis with pneumocephalus than an acute cerebrovascular event or transient ischemic attack. After multidisciplinary discussion among infectious disease, neurology, neurosurgery, otolaryngology, and critical care, the decision was made to transfer the patient to a tertiary facility for further specialized bilateral mastoidectomies.

## Discussion

Pneumocephalus occurs due to gas or air within the cranial cavity, with an overall low incidence rate of 0.5%-4.0% secondary to middle ear disease. It is most commonly associated with head and neck trauma, surgery, infection, and neoplasm [7]. In the clinical picture of pneumocephalus relating to mastoiditis, early symptoms include signs of intracranial pressure such as headache, vomiting, and papilledema and can extend to cranial nerve palsies. Rarely, focal neurological deficits that resemble cerebrovascular events may follow when the infection is severe. There is minimal literature regarding the presentation of erosive mastoiditis with pneumocephalus as an acute cerebrovascular event. A possible mechanism to explain this phenomenon may be due to a bone defect between the middle ear and cranium, allowing air to enter the intracranial cavity [8,9]. However, in our patient, no previous CT brain image was available for comparison, and the family denied such a defect as well as any antecedent trauma in the initial history taking.

Although middle ear infections resulting in intracranial pathology only account for a small number of cases, 5%-26% result in mortality [10]. Surgical management of such cases includes mastoidectomy, a procedure that removes thin bony particles of the temporal bone to open and release the postauricular air cells. Each procedure is highly unique and specialized due to the variety in size and shape of the temporal bone in aging adults as well as the extent of the disease. When operative management is not an option, conservative management with broad-spectrum antibiotics is the mainstay of treatment [11]. At this stage, urgent diagnosis and treatment are imperative for clinical resolution and to reduce mortality.

## Conclusions

Pneumocephalus is a rare complication of mastoiditis that can present with focal neurological deficits, deterring the main diagnosis. In the existing literature, there are a limited amount of cases describing such phenomena. A multidisciplinary approach to the evaluation and management is imminent to reduce mortality.
